# Pathophysiological Links Between Obesity and Dementia

**DOI:** 10.1007/s12017-023-08746-1

**Published:** 2023-04-22

**Authors:** David E. Wong Zhang, Vivian Tran, Antony Vinh, Quynh Nhu Dinh, Grant R. Drummond, Christopher G. Sobey, Maria Jelinic, T. Michael De Silva

**Affiliations:** Department of Microbiology, Anatomy, Physiology and Pharmacology, Centre for Cardiovascular Biology and Disease Research, La Trobe University, Bundoora, VIC 3086 Australia

**Keywords:** Obesity, Inflammation, Dementia, Gut-brain axis, Cognitive function

## Abstract

Obesity is a major global health concern, with prevalence rates rapidly rising due to increased availability of highly processed foods rich in fats and/or sugars and technological advances promoting more sedentary behaviour. There is increasing evidence to suggest that obesity predisposes individuals to developing cognitive impairment and dementia. However, the relationship between the brain and the peripheral metabolic state is complex, and many of the underlying mechanisms of cognitive impairment in obesity are yet to be fully elucidated. To better understand the links between obesity and dementia, further work is required to determine pathological changes occurring in the brain during obesity. In this mini-review, we discuss the role of two pathological features of obesity (the gut-brain axis and systemic inflammation) and their potential contribution to dementia.

## Introduction

Dementia describes a collection of conditions associated with severe and/or chronic cognitive impairment. Cognitive impairment is characterised by a deficit in at least one or more of the six cognitive domains: language, memory, attention, social/emotional, executive and visuospatial functioning. Several factors including aging, genetics, cardiovascular and metabolic health can modify the risk of developing dementia. Alarmingly, due to the aging population and that dementia primarily impacts individuals > 60 years of age, the prevalence of dementia is expected to rise from approximately 57 million global cases in 2019 to > 150 million in 2050 (Nicholas et al., 2022). At present there is a lack of effective therapies for dementia patients. A greater understanding of the pathophysiology of dementia, including contributing risk factors, will likely accelerate the development of new therapies.

Obesity is one of the fastest growing risk factors for the development of dementia. Body mass index (BMI) is the most commonly used standard in identifying overweight and obesity. The most widely reported BMI cut-offs are > 25 kg/m^2^ for overweight individuals and > 30 kg/m^2^ for obese (note, BMI cut-offs vary for different racial and ethnic categories). Approximately 1.2 billion individuals are classified as overweight, and a further 650 million people categorised as obese (World Health Organization, 2021). Obesity is a multifactorial disease characterised by the excessive accumulation and expansion of adipose tissue. While the pathology of obesity has significant lifestyle, behavioural and environmental components, we now appreciate that genetics also play a major role. Genetics are thought to account for 40–70% of the heritability of obesity (Loos & Yeo, 2022). Interestingly, obesity has a heterogeneous effect on dementia risk at different life stages (i.e. middle- vs late-adulthood; Singh-Manoux et al., 2018).

**Fig. 1:**
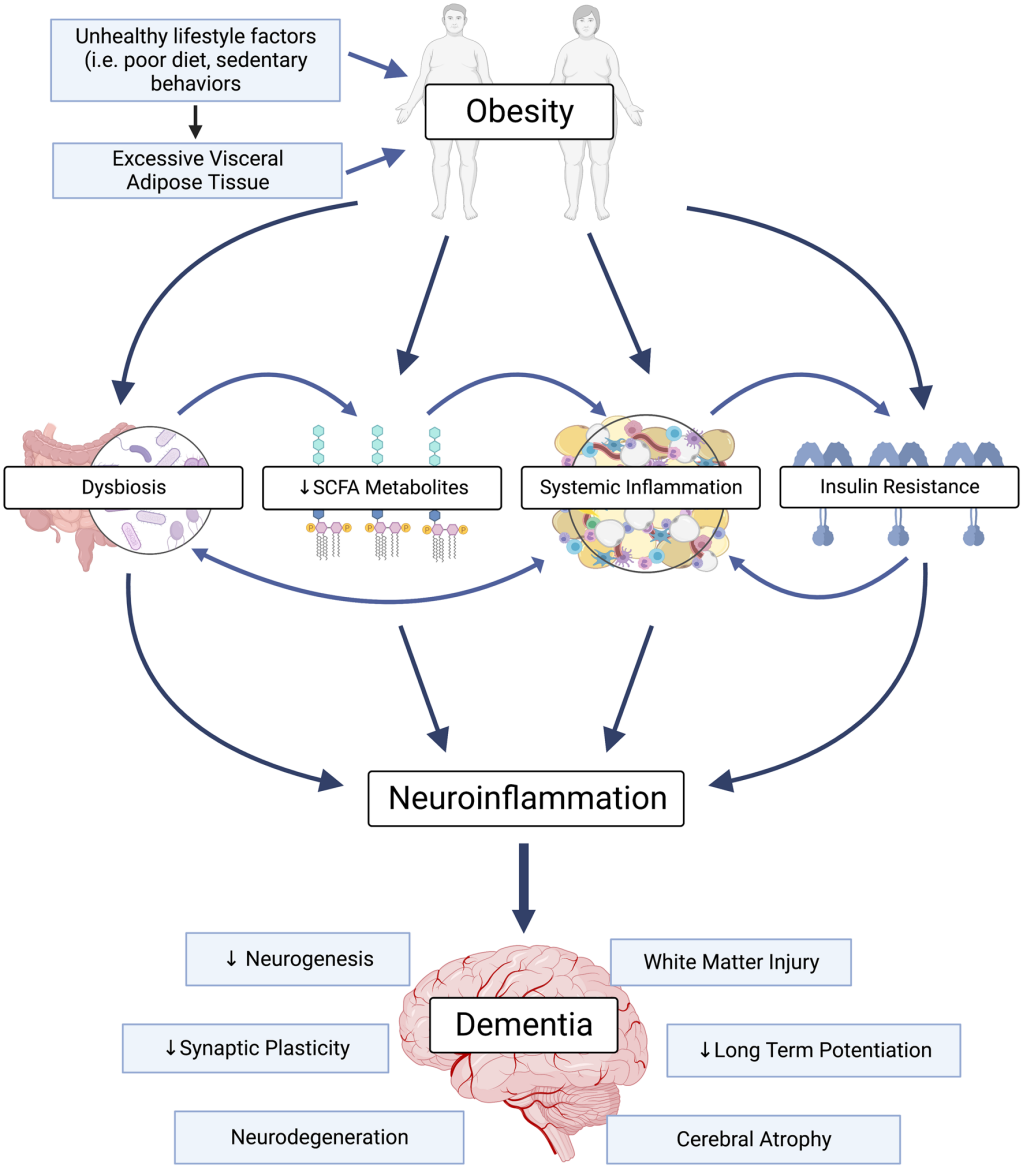
Obesity and the potential pathways to dementia. The complex interplay between obesity, neuroinflammation and dementia. Unhealthy lifestyle factors contribute to excess visceral adipose accumulation in obesity, which promotes systemic inflammation and insulin resistance. Obesity is also associated with gut dysbiosis and reduced short chain fatty acid (SCFA) metabolites. These pathological features promote neuroinflammation which in turn promotes the pathological features of dementia (i.e. decreased neurogenesis, synaptic plasticity and long-term potentiation; the development of white matter lesions; neurodegeneration and cerebral atrophy). Ultimately, this may impair cognition and cause dementia. Created with Biorender.com

While excess storage of nutrients is a hallmark of obesity, several other pathological features have been identified. Obesity promotes chronic, low-grade inflammation and hypertrophy of adipose tissue, which stimulates the release of pro-inflammatory mediators, resulting in oxidative stress and end-organ damage (Ellulu et al., 2017). Many of these pathological features have damaging effects throughout the body, including the brain. For example, inflammation and oxidative stress can compromise the precise delivery of oxygen and nutrients to active neurons (known as neurovascular coupling), which may lead to neuronal dysfunction. Impaired neurovascular coupling is a contributing factor in impaired cognitive function and dementia. The complexity of obesity and cognitive impairment makes identifying therapeutic targets to prevent or reverse disease challenging. In this mini-review we highlight some known pathological features of obesity (the gut-brain axis and systemic inflammation) which contribute to neuroinflammation and thus, may contribute to dementia (Fig. 1).

## Pathological Features of Obesity

Obesity results in a plethora of deleterious pathological features. These include insulin resistance (Kothari et al., 2017), gut dysbiosis (Saiyasit et al., 2020), oxidative stress (Hajiluian et al., 2018), inflammasome activation (Guo et al., 2020) and systemic inflammation (Wu et al., 2007) each of which may contribute to neuroinflammation and brain injury. For example, insulin resistance has been previously shown to contribute to neuroinflammation, neurodegeneration, and cognitive impairment (Kothari et al., 2017). Furthermore, hippocampal glutathione expression, a marker of oxidative stress and predictor of cognitive impairment, is significantly elevated people at risk of developing dementia (Turner et al., 2021).

Neuroinflammation is a well-established feature of dementia (Pasqualetti et al., 2015). Adipose tissue from obese individuals releases immune and/or metabolic mediators (e.g. C-reactive protein [CRP], interleukin [IL] -1β, leptin) that can cross the blood–brain barrier (BBB) and promote neuroinflammation (Van Dyken & Lacoste, 2018). These mediators can impair brain function leading to cognitive impairment and ultimately dementia (Van Dyken & Lacoste, 2018). Visceral adipose tissue also acts as an immunoendocrine organ secreting hormones, growth factors, enzymes and adipokines that regulate metabolism, mediate inflammation, and maintain body homeostasis (Kang et al., 2016). Adipokines that modulate insulin resistance, dysregulate the gut-brain axis, and increase systemic inflammation are of particular interest, as they are likely to contribute to neuroinflammation and the development of dementia pathology.

## The Gut-Brain Axis

Dementia progression may not only be influenced by intrinsic factors of the brain but also extrinsic factors from the gut. The gut-brain axis describes interactions between the gut microbiota, enteric nervous system (ENS) and central nervous system (CNS). Oxidative stress, driven by disrupted gut microbiota patterns, has been previously shown to be one of the key mechanisms promoting neuroinflammation (Loffredo et al., 2020). Determining the precise contribution of the gut microbiome to dementia is complex and requires further investigation as dysbiosis (altered gut microbiota patterns) also impacts metabolic, cardiovascular, gastrointestinal, and neuroimmune pathologies (Vamanu & Rai, 2021), each of which can contribute to the development of dementia. Nevertheless, it has been established that the gut and brain have a dynamic relationship in the development of cognitive impairment via neural, endocrine, metabolic, and immune pathways during dysbiosis (Forsythe et al., 2014). The gastrointestinal tract is regulated locally by the ENS and communicates with the CNS via the vagus nerve. Vagal afferent endings throughout the gastrointestinal tract can differentiate between pathogenic and non-pathogenic bacteria (Tanida et al., 2005). Interestingly, bacterial endotoxins, IL-1β, (Wieczorek et al., 2005) and TNF-α (Zanos et al., 2018) can send sensory input information via the vagal afferent fibres to the CNS to affect behaviour and impair cognitive function.

Gut microbiota can also alter gene expression, neurotransmitter release, receptor function and concentrations of metabolites within the digestive tract that are vital for the bidirectional communication between the gut and brain (Salami, 2021). For example, gut dysbiosis can alter tryptophan metabolism which affects serotonergic signalling (Whiley et al., 2021). Additionally, the loss of serotonergic inhibitory tone increases hypothalamic–pituitary–adrenal (HPA) activity in dementia patients (Whiley et al., 2021). Increased HPA activity also elevates cortisol levels to potentially induce hippocampal atrophy and impair cognition (Huijbers et al., 2020).

Short-chain fatty acid (SCFA) production by the gut microbiota is heavily influenced by diet and can alter a variety of metabolic pathways. In a healthy setting, SCFAs regulate energy homeostasis, induce anti-inflammatory functions, stimulate leptin production, and increase serotonin secretion (Liu et al, 2020). SCFAs derived from the gut microbiota may directly communicate with the brain via endotoxin translocation or vagus nerve stimulation/activation. Alternatively, SCFAs may signal to the brain indirectly via the actions of peptide hormones (Christiansen et al., 2018) or cytokines (Mirmonsef et al., 2012). Gut dysbiosis decreases SCFA levels which not only attenuates anti-inflammatory functions but also alters neuronal signalling which may impair cognitive function (Salami, 2021).

## Systemic Inflammation

It is well-established that systemic inflammation can promote neuroinflammation. Chronic systemic inflammation is a hallmark of obesity and can be instigated by adipose tissue expansion (adipocyte hypertrophy and proliferation). Adipose tissue expansion promotes a hypoxic environment where adipocytes undergo apoptosis, causing further inflammation (Lindhorst et al., 2021). Increased circulating pro-inflammatory cytokines and adipokines (e.g. TNF-α, leptin, resistin, plasminogen activator inhibitor-1, CRP, IL-1β and IL-6) contribute to systemic inflammation in obesity but are also established predictors of cognitive impairment (Kiliaan et al., 2014).

In obesity, adipokine release is dysregulated due to unhealthy adipose tissue expansion. This induces a phenotypic switch of adipose tissue type, increasing pro-inflammatory immune cells and altering the profiles of secreted adipokines. Indeed, adipose tissue from obese individuals release proportionally more pro-inflammatory adipokines compared to lean individuals, who secrete mainly anti-inflammatory adipokines (Wu et al., 2011). Leptin is a well-known adipokine that promotes neuronal health. Leptin also regulates energy intake and body mass. Additionally, elevated leptin levels increase TNF-α and IL-6 secretion from B cells, which further contributes to the pro-inflammatory state (Agrawal et al., 2011). High leptin levels can also promote leptin resistance and dysregulation of energy intake and body mass. Moreover, triglycerides can cross the BBB to directly induce hypothalamic leptin and insulin receptor resistance, which contributes to cognitive impairment (Banks et al., 2004). Inflammation in obesity disrupts the leptin signalling pathway to inhibit its protective effects on the brain (Forny-Germano et al., 2019). Importantly, leptin is regulated in a sex-dependent manner with greater circulating levels in females (Couillard et al., 1997). This sex-dependent regulation of leptin may contribute to the sexual dimorphisms in obesity and cognition, however, this requires further investigation.

Adiponectin is another adipokine of interest as it also regulates neuronal health. Additionally, it regulates glucose and fatty acid metabolism in the periphery (Yamauchi et al., 2002). Adiponectin can modify cytokines in the brain to exert anti-inflammatory and anti-oxidative effects (Thundyil et al., 2012). However, during obesity circulating adiponectin decreases which contributes to insulin resistance and a chronic pro-inflammatory state, making the body more susceptible to the development of cardiometabolic diseases (Forny-Germano et al., 2019).

The BBB is a semipermeable structure that facilitates the bidirectional movement of cells and circulating factors in the brain. Leptin and adiponectin can cross the BBB and have protective effects on the brain. However, in chronic inflammatory conditions, BBB integrity is compromised, allowing potentially neurotoxic substances access to the brain. Other circulating factors released in obesity, such as IL-6 and TNF-α, further increase BBB permeability (Rochfort et al., 2016). Moreover, IL-1β and IL-6 can directly bind to neurons within the hippocampus to cause neuronal dysfunction and impair working memory (Dinel et al., 2011). In addition, TNF-α and its receptor (TNF receptor 1) are necessary for synaptic disruptions in astrocytes (Habbas et al., 2015). Increased TNF-α alters the excitatory transmission in the hippocampal cognitive circuit which contributes to learning and memory deficits (Habbas et al., 2015). The collective damage to the brain of such pro-inflammatory molecules promotes cerebral atrophy and white matter injury, ultimately increasing the risk of dementia.

## Linking Obesity and Dementia

The pathological changes discussed above which contribute to neuroinflammation likely precede dementia by many years. If not addressed, obesity may result in irreversible damage to the brain which only manifests as a clinical diagnosis later in life. Indeed, while the classification of obesity as a risk factor for dementia has been somewhat controversial, recent work has revealed mid-life obesity as the critical timepoint for increased risk of developing dementia later in life. Longitudinal studies have revealed that having a BMI > 25 was associated with increased risk of developing dementia 10–15 years later (Bowman et al., 2019; Singh-Manoux et al., 2018). The association was lost when individuals were obese closer to dementia diagnosis (Singh-Manoux et al., 2018). Conversely, weight loss late in life (likely due to malnutrition) is associated with an increased risk of dementia (Joo et al., 2018). Thus, as with other risk factors for dementia (such as hypertension), being obese in mid-life appears to be the critical timeframe for increased disease risk. At this point in the disease process, the pathological features of obesity likely causes long lasting damage to the brain. While not immediately apparent, the damage to the brain parenchyma may ultimately lead to dementia later in life.

## Conclusion

In this mini-review, we have highlighted some of the evidence for the pathological features of obesity that may contribute to brain injury and ultimately dementia. However, there remains major knowledge gaps regarding the contribution of obesity to dementia. Further investigation is needed to elucidate the specific mechanisms that underly obesity-induced dementia. A better understanding of dementia pathology and its mechanisms in the setting of obesity is critical for the development of better treatments for the rapidly expanding number of patients with these debilitating conditions.
